# Macular perfusion alterations in people with recent-onset diabetes and novel diabetes subtypes

**DOI:** 10.1007/s00125-025-06407-5

**Published:** 2025-03-31

**Authors:** Sema Kaya, Ala Khamees, Gerd Geerling, Piotr Strzalkowski, Veronika Gontscharuk, Julia Szendroedi, Karsten Müssig, Dan Ziegler, Michael Roden, Rainer Guthoff, for the GDS Cohort

**Affiliations:** 1 Department of Ophthalmology, Medical Faculty and University Hospital Düsseldorf, Heinrich Heine University Düsseldorf, Düsseldorf, Germany; 2 Institute for Health Services Research and Health Economics, German Diabetes Center, Leibniz Center for Diabetes Research at Heinrich Heine University Düsseldorf, Düsseldorf, Germany; 3 Institute for Health Services Research and Health Economics, Centre for Health and Society, Medical Faculty and University Hospital Düsseldorf, Heinrich Heine University Düsseldorf, Düsseldorf, Germany; 4 German Center for Diabetes Research (DZD e.V.), München-Neuherberg, Germany; 5 Department of Internal Medicine I, Medical Faculty, Ruprecht Karls University Heidelberg, Heidelberg, Germany; 6 Department of Internal Medicine, Gastroenterology and Diabetology, Niels Stensen Hospitals, Franziskus Hospital Harderberg, Georgsmarienhütte, Germany; 7 Institute for Clinical Diabetology, German Diabetes Center, Leibniz Center for Diabetes Research at Heinrich Heine University Düsseldorf, Düsseldorf, Germany; 8 Division of Endocrinology and Diabetology, Medical Faculty and University Hospital Düsseldorf, Heinrich Heine University Düsseldorf, Düsseldorf, Germany

**Keywords:** Choriocapillaris, Diabetes subtypes, Diabetic retinopathy, Foveal avascular zone, Ganglion cell layer, Microaneurysms, Microvasculopathy, OCTA, Perfusion density, Vessel density

## Abstract

**Aims/hypothesis:**

Our aim was to detect early structural and functional changes in the macular capillaries using optical coherence tomography angiography during the course of type 1 or 2 diabetes mellitus.

**Methods:**

In this cross-sectional study, individuals with type 1 diabetes (*n*=143) or type 2 diabetes (*n*=197) from the German Diabetes Study (ClinicalTrials.gov registration no. NCT01055093) underwent clinical examination and cluster analysis to identify phenotype-based diabetes subtypes, using BMI, age, HbA_1c_, homoeostasis model estimates and islet autoantibodies. Colour fundus photography, optical coherence tomography and optical coherence tomography angiography were performed within the first year of diabetes diagnosis (baseline) and at 5 year intervals up to year 10. Age- and sex-adjusted participants served as control participants (*n*=105). Perfusion density, vessel density, presence of retinal microaneurysms in superficial, intermediate and deep capillary plexus (SCP, ICP, DCP), choriocapillaris flow deficit density (CC FD) and the foveal avascular zone (FAZ) of the macula as well as retinal layer thickness, visual acuity and contrast sensitivity were analysed.

**Results:**

Perfusion density and vessel density of SCP were already reduced at baseline in type 2 diabetes (expected difference compared with control participants: −0.0071, *p*=0.0276, expected difference: −0.0034, *p*=0.0184, respectively), especially in participants with severe insulin-deficient and mild obesity-related diabetes. At year 10 only perfusion density of the SCP and DCP was reduced in both type 1 and 2 diabetes (*p*=0.0365, *p*=0.0062, respectively). The FAZ was enlarged and the CC FD within the first year increased in type 1 (*p*=0.0327, *p*=0.0474, respectively) and more markedly in type 2 diabetes (*p*=0.0006, *p*<0.0001). The occurrence of microaneurysms in SCP and DCP was significant at year 5 (*p*=0.0209, *p*=0.0279, respectively) and year 10 (*p*=0.0220, *p*=0.0007). Presence of microaneurysms in SCP and DCP was associated with decreases in perfusion density and vessel density in both SCP and ICP. Furthermore, microaneurysms were associated with decreased ganglion cell layer and inner plexiform layer thickness.

**Conclusions/interpretation:**

Type 2 diabetes already reduces macular perfusion SCP at time of clinical diagnosis, while long-standing diabetes affects both SCP and DCP. The FAZ of the SCP and the CC FD are early indicators of diabetic alterations, with more pronounced changes observed in type 2 diabetes. Microaneurysms in the macular plexus are associated with a decrease of ganglion cell layer and inner plexiform layer. Subclinical microangiopathy occurs prior to manifestation of diabetic retinopathy, disease-related visual acuity impairment or inner retinal layer thinning.

**Graphical Abstract:**

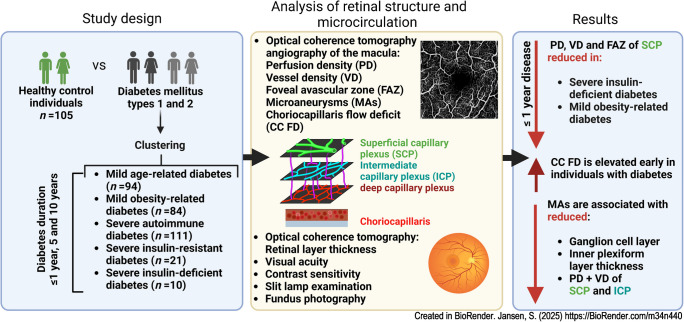

**Supplementary Information:**

The online version of this article (10.1007/s00125-025-06407-5) contains peer-reviewed but unedited supplementary material.


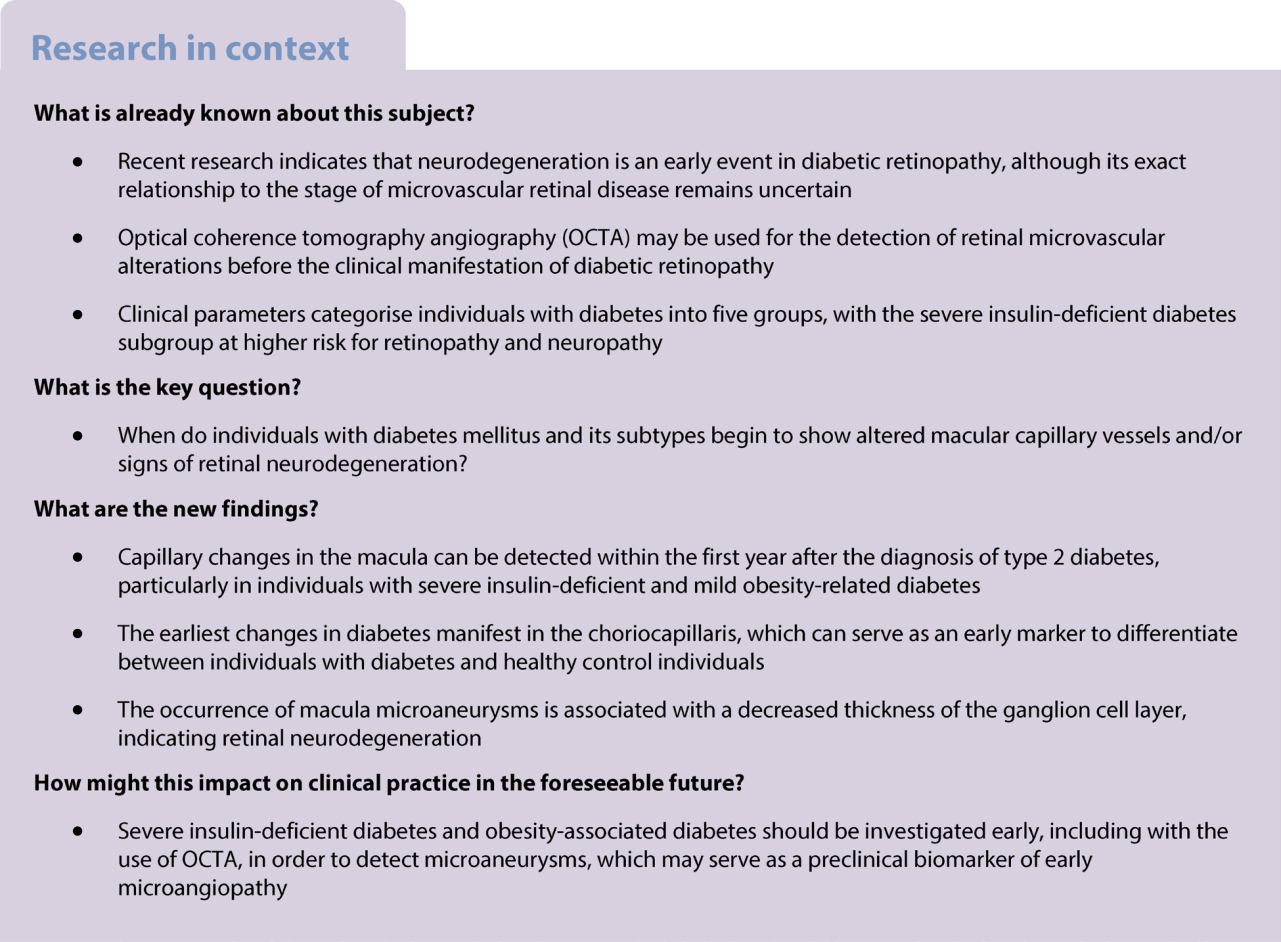


## Introduction

Diabetic retinopathy, one of the most common complications of diabetes mellitus, currently affects 100 million people worldwide and is responsible for severe visual impairment and blindness [1]. Clinically, diabetic retinopathy is characterised by microvascular dysfunction manifesting in retinal microaneurysms (MAs), haemorrhages, macular oedema with hard exudates and retinal neovascularisation [2]. Diagnosis and staging are performed using ophthalmoscopy under pupil dilation and, if feasible, documented with fundus photography [3].

Microvascular changes may be detectable with optical coherence tomography angiography (OCTA) before the appearance of MAs, the first fundoscopically visible sign of diabetic retinopathy [4]. OCTA non-invasively uses erythrocyte movement as a contrast to distinguish perfused vessels from static tissue and to quantify preclinical abnormalities in the retinal vasculature [5]. Whether and to what extent preclinical diabetic retinal changes can be detected by OCTA is currently controversial [6, 7].

According to current understanding, diabetic retinopathy evolves as an interplay between diabetic microangiopathy and diabetic retinal neurodegeneration [8]. Diabetic neurodegeneration refers to the decline in visual function in diabetes, characterised by retinal apoptosis, glial activation, reduced inner retinal thickness on optical coherence tomography (OCT) and functional deficits such as decreased amplitudes, delayed implicit times and reduced retinal sensitivity [9]. The temporal relationship between vascular and neural components in diabetes remains unclear. While some studies suggest that neurodegeneration precedes vascular changes, others propose independent effects of diabetes on both, leaving their exact interplay unresolved [10, 11]. The prospective observational German Diabetes Study (GDS) [12] allows early capillary and neurodegenerative changes to be analysed using a cohort that has been comprehensively phenotyped in the first year of diabetes diagnosis and at follow-up examinations for up to 15 years. Our study aims to determine when individuals with diabetes mellitus or its subtypes begin to show altered macular capillary vessels and/or signs of retinal neurodegeneration.

## Methods

### Study population

The GDS (ClinicalTrials.gov registration no. NCT01055093) performs comprehensive phenotyping for metabolic alterations and the development of comorbidities and complications during the course of diabetes [12]. The inclusion and exclusion criteria have been described in detail [12]. Individuals with diabetes were cluster analysed for phenotype-based subtype determination, following sex-specific classification criteria of Ahlqvist and colleagues [13]. When the study was designed, it was decided to record participants’ sex through self-report and only include people of European descent, to exclude effects of genetic variability. Each participant was assigned to one of the predefined clusters: mild age-related diabetes (MARD), mild obesity-related diabetes (MOD), severe insulin-deficient diabetes (SIDD), severe insulin-resistant diabetes (SIRD) or severe autoimmune diabetes (SAID), which aligns with type 1 diabetes [14]. For this analysis, the clusters were age-adjusted to exclude age-related effects. For the cross-sectional analyses at baseline, we used a consecutive sample with all participants from the ongoing GDS who had complete baseline data for the allocation to diabetes subtypes and who had undergone a complete ophthalmic examination including OCTA. Tables 1 and 2 show the enrolled study population. The GDS, conducted in accordance with the Declaration of Helsinki, was approved by the local institutional ethics committee (reference number 4508). Informed written consent was obtained from all participants.

**Table 1:** Demographic and clinical characteristics of participants with type 1 and type 2 diabetes and control participants

Characteristic	T1DM (*N*=143)	T2DM (*N*=197)	*p* value^a^	Control participants (*N*=105)	*p* value^b^	*p* value^c^	*p* value^d^
Age (years)	41.2 ± 12.0	57.6 ± 10.3	<0.0001	46.5 ± 15.3	0.0053	<0.0001	<0.0001
Male sex	77 (53.8)	132 (67.0)	0.0176	58 (55.2)	0.8975	0.0464	0.0263
BMI (kg/m^2^)	25.7 ± 4.4	31.5 ± 6.4	<0.0001	26.8 ± 5.1	0.0798	<0.0001	<0.0001
HbA_1c_ (mmol/mol)	51.3 ± 10.2	51.1 ± 13.0	0.3512	33.1 ± 3.6	<0.0001	<0.0001	<0.0001
HbA_1c_ (%)	6.8 ± 0.9	6.8 ± 1.2	0.3512	5.2 ± 0.3	<0.0001	<0.0001	<0.0001
Blood pressure systolic/diastolic (mmHg)	128.7 ± 16.9/79.8 ± 9.6	139.9 ± 16.7/84.7 ± 10.0	<0.0001/<0.0001	131.4 ± 16.2/80.5 ± 10.7	0.1253/0.7392	<0.0001/0.0005	<0.0001/<0.0001
Known diabetes duration in years (*n*)	BL: 52 (36.4) Y5: 51 (35.7) Y10: 39 (27.3) Y15: 1 (0.7)	BL: 65 (33.0) Y5: 71 (36.0) Y10: 58 (29.4) Y15: 3 (1.5)	0.8159^e^				
NPDR (*n*)	3 (2.1)	6 (3.0)	0.7391				

Data are presented as mean ± SD for quantitative variables and *n* (%) for categorical variables

^a^Comparison of T1DM and T2DM

^b^Comparison of T1DM and control participants

^c^Comparison of T2DM and control participants

^d^Comparison of T1DM, T2DM and control participants. In the case of quantitative variables, *p* values were calculated via Kruskal–Wallis test for comparison of three groups (i.e. *p* value^d^) and Mann–Whitney *U* test for comparison of two groups (i.e. *p* value^a^, *p* value^b^ and *p* value^c^). For categorical variables, *p* values are related to χ^2^ test or Fisher’s exact test when it was possible

^e^*p* value was calculated without cases with Y15 since assumptions of χ^2^ test were not fulfilled for all four categories of diabetes duration

BL, baseline; NPDR, non-proliferative diabetic retinopathy; T1DM, type 1 diabetes; T2DM, type 2 diabetes; Y5–Y15, years of follow-up

**Table 2:** Demographic and clinical characteristics of different diabetes clusters

Characteristic	SAID (111)	SIDD (10)	SIRD (21)	MOD (84)	MARD (94)	*p* value^a^
Age (years)	41.6 ± 12.5	44.9 ± 12.3	61.0 ± 12.4	51.4 ± 9.4	60.8 ± 10.1	<0.0001
Male sex (%)	57 (51.4)	8 (80.0)	15 (71.4)	47 (56.0)	71 (75.5)	0.0029
BMI (kg/m^2^)	25.5 ± 4.2	26.6 ± 4.3	36.0 ± 5.5	34.9 ± 6.1	27.0 ± 3.8	<0.0001
Blood pressure (mmHg)	128.0 ± 17.5/80.0 ± 10.1	139.9 ± 13.7/86.7 ± 6.6	143.6 ± 16.6/85.5 ± 11.6	140.2 ± 16.0/87.1 ± 8.9	137.6 ± 17.9/81.6 ± 10.3	<0.0001/<0.0001
HbA_1c_ (mmol/mol)	51.4 ± 9.4	68.0 ± 14.2	47.9 ± 13.0	52.5 ± 13.9	47.9 ± 9.3	<0.0001
HbA_1c_ (%)	6.9 ± 0.9	8.4 ± 1.3	6.5 ± 1.2	7.0 ± 1.3	6.5 ± 0.8	<0.0001
NPDR (*n*)	3 (2.7)	1 (10.0)	0	3 (3.6)	1 (1.1)	0.3985^b^
Participants at the visits (*n*)	BL: 43 (38.7) Y5: 38 (34.2) Y10: 29 (26.1) Y15: 1 (0.9)	BL: 5 (50.0) Y5: 4 (40.0) Y10: 1 (10.0)	BL: 7 (33.3) Y5: 6 (28.6) Y10: 8 (38.1)	BL: 22 (26.2) Y5: 38 (45.2) Y10: 24 (28.6)	BL: 34 (36.2) Y5: 30 (31.9) Y10: 30 (31.9)	0.4033^c^

Data are presented as mean ± SD for quantitative variables and *n* (%) for categorical variables

^a^Comparison of the five diabetes clusters. In the case of quantitative variables, *p* values were calculated via Kruskal–Wallis test for comparison of the five clusters. For categorical variables, *p* values are related to χ^2^ test

^b^Assumptions of χ^2^ were not fulfilled

^c^*p* value was calculated without cases with Y15 since assumptions of χ^2^ test were not fulfilled for all four categories of diabetes duration

BL, baseline; NPDR, non-proliferative diabetic retinopathy; Y5–Y15, years of follow-up

The ophthalmological examinations were performed at the University Hospital of Ophthalmology Düsseldorf as described by Schröder et al [15]. Classification was based on the International Clinical Diabetic Retinopathy Disease Severity Scale [16]. Non-mydriatic wide-angle fundus documentation was performed (Zeiss Clarus 500, Carl Zeiss Meditec, Jena, Germany, Optos, Dunfermline, UK). The distribution of the included individuals for visual acuity (VA) and contrast sensitivity (CS, at reading distance using the Mars Letter Test, Mars Percetrix, Chappaqua, NY, USA) is shown in Table 3.

**Table 3:** ETDRS VA and CS in control participants and participants with type 1 and type 2 diabetes within 1 year after diagnosis (baseline) and 5 and 10 years after diagnosis

Variable	VA		CS		
	RE	LE	RE	LE	BE
Baseline					
Control	98 84.1 ± 8.3	98 83.8 ± 7.6	92 1.6 ± 0.1	89 1.6 ± 0.1	91 1.7 ± 0.1
T1DM	46 86.8 ± 5.4	44 85.9 ± 8.0	47 1.6 ± 0.1	46 1.6 ± 0.1	46 1.7 ± 0.1
T2DM	62 81.5 ± 12.2	61 84.1 ± 6.4	62 1.5 ± 0.1	60 1.6 ± 0.1	60 1.7 ± 0.1
5 years					
T1DM	46 87.2 ± 5.6	46 86.2 ± 7.3	45 1.6 ± 0.1	45 1.6 ± 0.1	45 1.7 ± 0.2
T2DM	65 82.2 ± 9.1	65 82.5 ± 7.8	66 1.5 ± 0.1	64 1.5 ± 0.2	65 1.7 ± 0.1
10 years					
T1DM	38 80.6 ± 15.2	37 82.8 ± 7.3	37 1.6 ± 0.3	35 1.6 ± 0.1	38 1.7 ± 0.1
T2DM	51 78.5 ± 14.6	49 80.4 ± 9.0	51 1.5 ± 0.1	49 1.5 ± 0.2	52 1.6 ± 0.1

Data are presented as sample size, mean ± SD

BE, both eyes; LE, left eye; RE, right eye; T1DM, type 1 diabetes; T2DM, type 2 diabetes

### OCTA protocol

OCTA and OCT images were acquired by a trained professional photographer using spectral domain OCT (Spectralis, Heidelberg Engineering, Germany). Foveally centred 512 A-scans × 512 B-scans covering a scan pattern of 10° × 10° (~2.9 × 2.9 mm) with a lateral resolution of 5.7 μm/pixel and an axial resolution of 3.9 μm/pixel were performed in enhanced depth imaging (EDI) mode. En face OCTA images of the superficial, intermediate and deep capillary plexus (SCP, ICP, DCP) and choriocapillaris with activated artefact removal mode were exported from the Spectralis Heidelberg Eye Explorer viewing program (software version 1.10.2.0, Heidelberg Engineering, Heidelberg, Germany) into the tagged image file format for analysis. The artefact rate was minimised by implementing measures to enhance participant comfort and stability, as well as improving repeatability with real-time feedback. Low-quality OCTA scans (23%, 32.8% for choriocapillaris) were excluded due to reduced resolution, motion artefacts, defocus or shadowing.

**Fig. 1:**
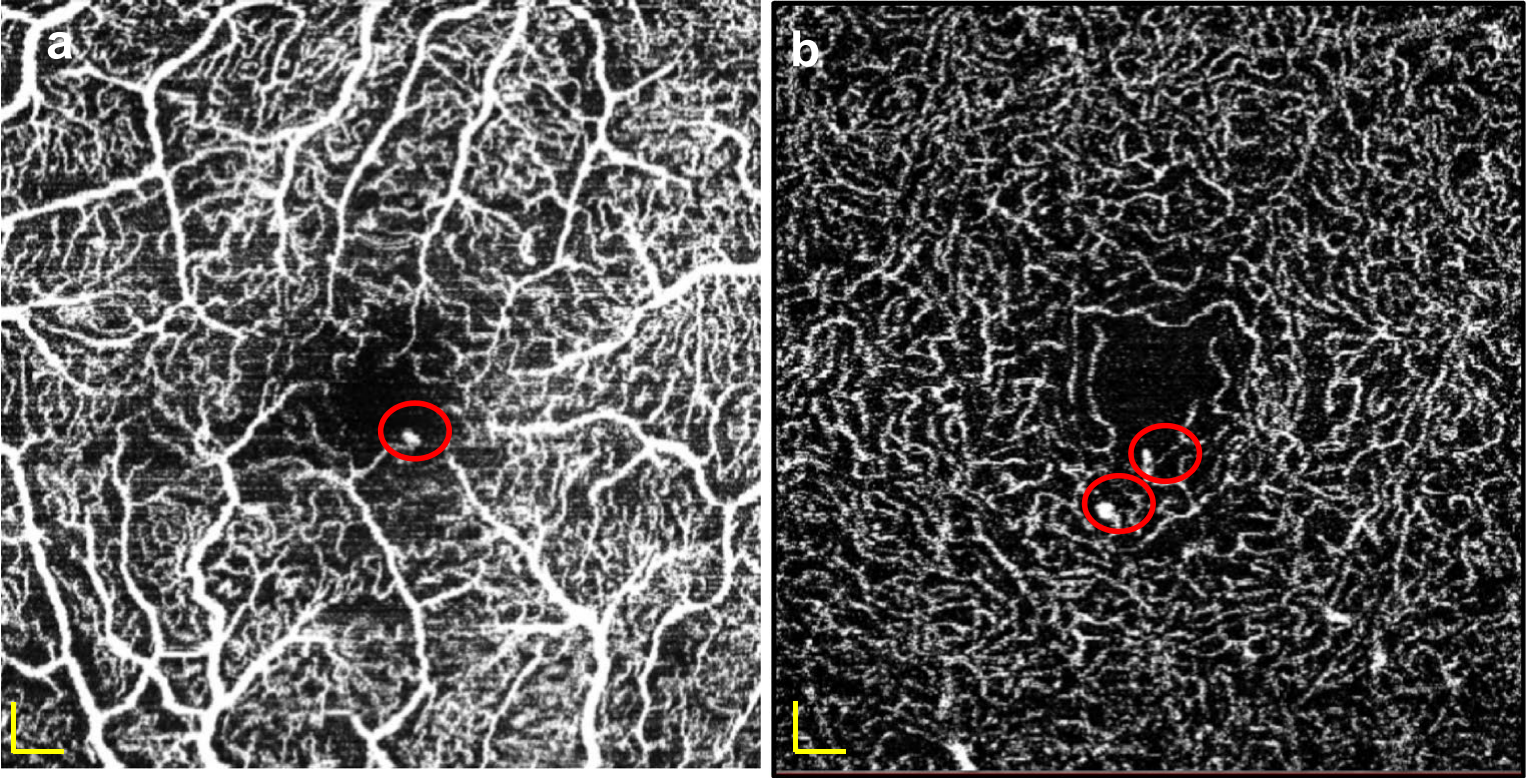
Retinal MAs. Examples of MAs (circled in red) of a participant with diabetes located in the SCP (**a**) and ICP (**b**) within the foveal-centred (10° × 10°) area. Scale bar, 200 μm

Images were generally blindly analysed. Two experienced ophthalmologists performed the detection of MAs in the foveal-centred area (Fig. 1).

The foveal avascular zone (FAZ, mm^2^) of the SCP was manually delineated by a trained clinical expert, while the FAZ perimeter (mm) was automatically measured, both using Fiji (ImageJ, version 1.53c; http://imagej.nih.gov/ij) [17]. FAZ circularity was calculated using the formula: circularity = 4π × (area)/(perimeter^2^), which quantifies the degree of similarity to a circle (with a value of 1 for a perfect circle, while 0 reflects an irregular shape) [18].

Choriocapillaris flow deficit density (CC FD) was analysed after binarisation using Phansalkar’s local thresholding method (6 pixel radius, 17.4 µm) and calculated as the flow deficit area divided by the total area using the Analyze Particles tool in ImageJ [19].

**Fig. 2:**
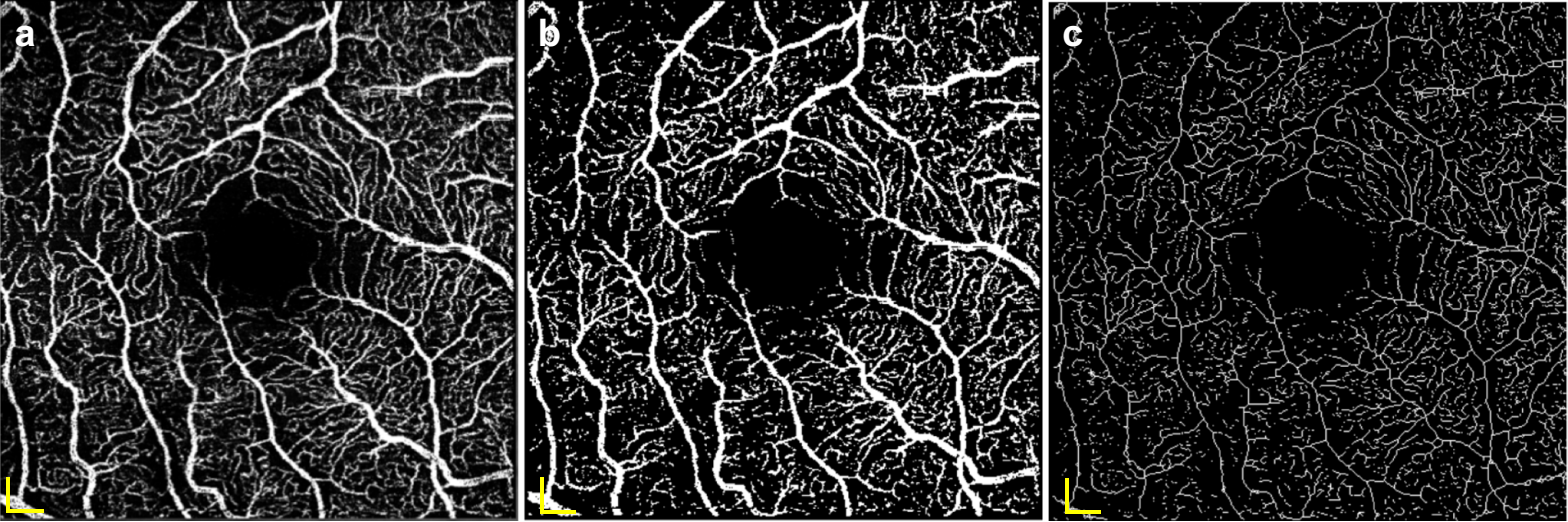
Visualisation of image processing. All OCTA images were converted into 8 bit files (**a**). Then, images were binarised using the Otsu thresholding method (**b**). The binarised images were used to calculate the PD, which is determined as the number of flow pixels divided by the total number of pixels. To calculate the VD, the influence of vessel diameter was eliminated by generating a skeletonised image (**c**). Then, the number of white pixels was divided by the total number of pixels. Scale bar, 200 μm

To calculate perfusion density (PD, defined as the percentage of perfused area, calculated by dividing the number of flow pixels by the total number of pixels), images were converted to 8 bit files using Fiji and binarised using the Otsu thresholding method. The vessel density (VD, the sum of vessel lengths per area) was obtained by dividing the number of white pixels by the number of total pixels after eliminating the influence of vessel diameter by skeletonising the vessels into a line 1 pixel wide (Fig. 2) [20].

For PD and VD analysis of the SCP, ICP and DCP, 333 eyes of 172 individuals with type 2 diabetes, 210 eyes of 107 individuals with type 1 diabetes and 185 eyes of 98 control individuals were available (Table 4). The distribution of the included participants for FAZ and CC FD is summarised in Tables 5 and 6.

**Table 4:** PD and VD of control participants and participants with type 1 and type 2 diabetes within 1 year of diagnosis (at baseline) and 5 and 10 years after diagnosis

Variable	VD of SCP			PD of SCP			PD of ICP			PD of DCP		
	RE	LE	Missing values	RE	LE	Missing values	RE	LE	Missing values	RE	LE	Missing values
Baseline												
Control, *N*=105	94 0.09 ± 0.01	91 0.09 ± 0.01	25 (11.90)	94 0.21 ± 0.03	91 0.21 ± 0.03	25 (11.90)	93 0.26 ± 0.03	91 0.26 ± 0.03	26 (12.38)	93 0.27 ± 0.04	90 0.23 ± 0.08	27 (12.86)
T1DM, *N*=52	34 0.09 ± 0.01	34 0.09 ± 0.01	36 (34.62)	34 0.22 ± 0.02	34 0.21 ± 0.02	36 (34.62)	34 0.27 ± 0.02	34 0.26 ± 0.03	36 (34.62)	34 0.27 ± 0.03	34 0.23 ± 0.07	36 (34.62)
T2DM, *N*=65	52 0.09 ± 0.01	55 0.09 ± 0.01	23 (17.69)	52 0.21 ± 0.03	55 0.20 ± 0.03	23 (17.69)	53 0.25 ± 0.03	55 0.25 ± 0.03	22 (16.92)	53 0.26 ± 0.04	55 0.24 ± 0.06	22 (16.92)
5 years												
T1DM, *N*=51	39 0.09 ± 0.01	40 0.09 ± 0.01	23 (22.55)	39 0.22 ± 0.02	40 0.21 ± 0.02	23 (22.55)	39 0.26 ± 0.03	40 0.26 ± 0.02	23 (22.55)	39 0.27 ± 0.04	40 0.23 ± 0.07	23 (22.55)
T2DM, *N*=71	62 0.09 ± 0.01	62 0.09 ± 0.01	18 (12.68)	62 0.20 ± 0.02	62 0.20 ± 0.02	18 (12.68)	62 0.24 ± 0.03	62 0.24 ± 0.03	18 (12.68)	63 0.25 ± 0.03	62 0.22 ± 0.06	17 (11.97)
10 years												
T1DM, *N*=39	32 0.09 ± 0.01	29 0.09 ± 0.01	17 (21.79)	32 0.20 ± 0.02	29 0.20 ± 0.02	17 (21.79)	33 0.24 ± 0.03	29 0.25 ± 0.03	16 (20.51)	33 0.25 ± 0.03	29 0.19 ± 0.07	16 (20.51)
T2DM, *N*=58	45 0.08 ± 0.01	48 0.08 ± 0.01	23 (19.83)	45 0.19 ± 0.03	48 0.19 ± 0.02	23 (19.83)	45 0.24 ± 0.04	48 0.24 ± 0.05	23 (19.83)	44 0.24 ± 0.03	48 0.19 ± 0.08	24 (20.69)

Data are presented as sample size, mean ± SD. Missing values are presented as both sample size and percentage

LE, left eye; RE, right eye; T1DM, type 1 diabetes; T2DM, type 2 diabetes

**Table 5:** FAZ area, FAZ perimeter and FAZ circularity of control participants and participants with type 1 and type 2 diabetes within 1 year of diagnosis (at baseline) and 5 and 10 years after diagnosis

Variable	FAZ area		FAZ perimeter		FAZ circularity		Missing values
	RE	LE	RE	LE	RE	LE	
Baseline							
Control, *N*=105	89 0.38 ± 0.15	85 0.37 ± 0.15	89 3.42 ± 1.16	85 3.29 ± 0.98	89 0.44 ± 0.16	85 0.46 ± 0.16	36 (17.14)
T1DM, *N*=52	34 0.42 ± 0.18	34 0.45 ± 0.20	34 3.93 ± 1.83	34 4.57 ± 2.81	34 0.42 ± 0.19	34 0.37 ± 0.17	36 (34.62)
T2DM, *N*=65	52 0.46 ± 0.16	52 0.47 ± 0.15	52 5.03 ± 2.14	52 5.30 ± 2.19	52 0.31 ± 0.19	52 0.28 ± 0.17	26 (20.00)
5 years							
T1DM, *N*=51	41 0.41 ± 0.16	41 0.41 ± 0.16	41 4.11 ± 1.73	41 4.28 ± 1.69	41 0.36 ± 0.17	41 0.34 ± 0.18	20 (19.61)
T2DM, *N*=71	63 0.45 ± 0.15	60 0.45 ± 0.12	63 4.57 ± 1.82	60 4.74 ± 1.63	63 0.33 ± 0.16	60 0.31 ± 0.16	19 (13.38)
10 years							
T1DM, *N*=39	33 0.42 ± 0.16	31 0.45 ± 0.21	33 4.24 ± 1.96	31 4.32 ± 1.69	33 0.37 ± 0.17	31 0.36 ± 0.16	14 (17.95)
T2DM, *N*=58	49 0.45 ± 0.14	46 0.44 ± 0.15	49 4.30 ± 1.43	46 4.32 ± 1.48	49 0.36 ± 0.17	46 0.35 ± 0.17	21 (18.10)

Data are presented as sample size, mean ± SD. Missing values are presented as both sample size and percentage. FAZ area is given in mm^2^ and perimeter in mm

LE, left eye; RE, right eye; T1DM, type 1 diabetes; T2DM, type 2 diabetes

**Table 6:** Choriocapillaris flow deficit area of control participants and participants with type 1 and type 2 diabetes within 1 year of diagnosis (at baseline) and 5 and 10 years after diagnosis

Variable	Choriocapillaris flow deficit area (%)		Missing values
	RE	LE	
Baseline			
Control, *N*=105	74 38.03 ± 7.01	71 37.44 ± 6.85	65 (30.95)
T1DM, *N*=52	31 36.85 ± 5.63	31 37.77 ± 6.25	42 (40.38)
T2DM, *N*=65	50 44.50 ± 8.98	50 44.36 ± 7.48	30 (23.08)
5 years			
T1DM, *N*=51	34 38.22 ± 6.08	32 39.83 ± 7.44	36 (35.29)
T2DM, *N*=71	58 44.80 ± 6.87	51 45.50 ± 7.73	33 (22.24)
10 years			
T1DM, *N*=39	28 40.61 ± 5.70	24 40.84 ± 6.51	26 (33.33)
T2DM, *N*=58	43 45.58 ± 8.04	42 45.99 ± 8.47	31 (26.72)

Data are presented as sample size, mean ± SD. Missing values are presented as both sample size and percentage

LE, left eye; RE, right eye; T1DM, type 1 diabetes; T2DM, type 2 diabetes mellitus

### OCT protocol

**Fig. 3:**
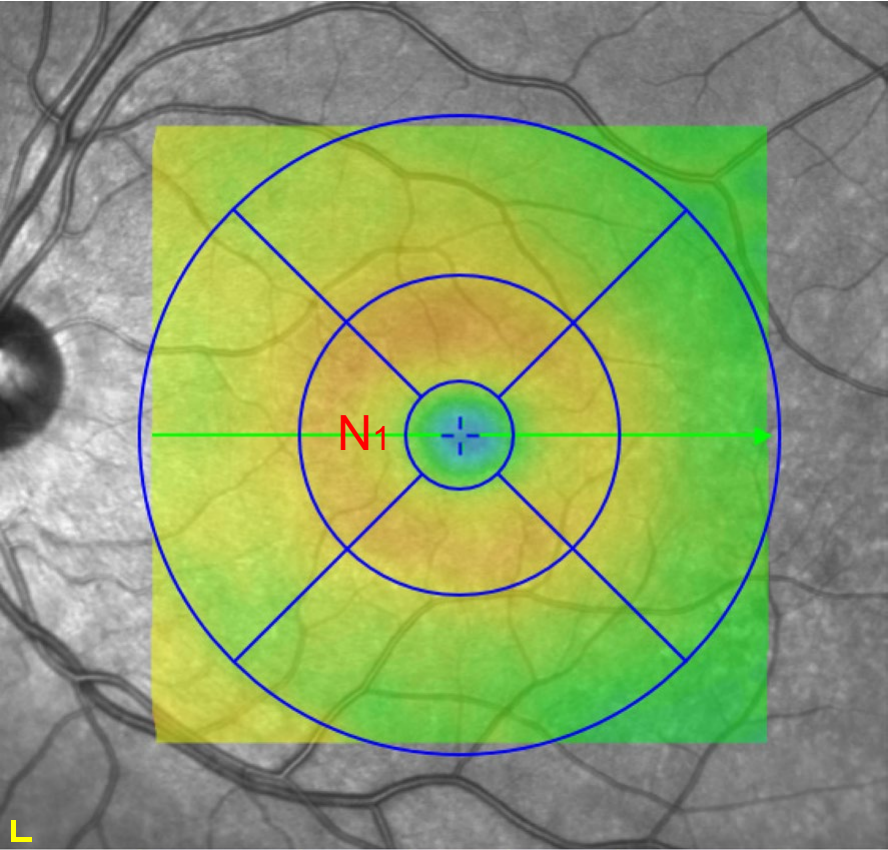
Spectral domain coherence tomography thickness map. Spectral domain OCT thickness map labelling the 1-, 3- and 6-mm ETDRS subfield, with the examined nasal pericentral segment N1 highlighted. Scale bar, 200 μm

For retinal layer thickness analysis, retinal layers were segmented using the Spectralis Heidelberg Eye Explorer software and subsequently screened for segmentation errors. The thicknesses of full retina, retinal nerve fibre layer (RNFL), ganglion cell layer (GCL), inner plexiform layer (IPL), inner nuclear layer (INL), outer plexiform layer (OPL), outer nuclear layer (ONL) and retinal pigment epithelium (RPE) were determined in the inner nasal pericentral segment of the foveal-centred Early Treatment Diabetic Retinopathy Study (ETDRS) grid, the site of importance for full central VA and CS [15] (Fig. 3). Only essential parameters were analysed to avoid multiple testing, as summarised in Table 7.

**Table 7:** Full retinal and retinal layer thickness of control participants and participants with type 1 and type 2 diabetes within 1 year after diagnosis (baseline) and 5 and 10 years after diagnosis in RNFL, GCL, IPL, INL, OPL, ONL and RPE

Variable	Full retinal thickness		RNFL		GCL		IPL		INL		OPL		ONL		RPE	
	RE	LE	RE	LE	RE	LE	RE	LE	RE	LE	RE	LE	RE	LE	RE	LE
Baseline																
Control	102 349.7 ± 17.1	101 349.5 ± 17.0	102 21.6 ± 2.2	101 21.4 ± 2.4	102 52.4 ± 5.4	101 52.9 ± 5.6	102 43.8 ± 4.1	101 43.7 ± 3.9	102 42.2 ± 3.6	101 42.1 ± 3.6	102 32.8 ± 6.4	101 33.6 ± 7.1	102 74.2 ± 11.9	101 73.0 ± 11.0	102 15.6 ± 1.8	101 15.6 ± 1.8
T1DM	47 346.4 ± 14.5	46 346.3 ± 14.4	47 21.2 ± 2.1	46 21.2 ± 2.4	47 51.6 ± 5.4	46 51.4 ± 5.3	47 42.8 ± 3.6	46 42.8 ± 3.7	47 41.5 ± 4.1	46 42.1 ± 4.4	47 33.0 ± 7.8	46 33.3 ± 8.2	47 73.1 ± 10.9	46 72.6 ± 10.5	47 15.8 ± 1.9	46 15.7 ± 1.7
T2DM	60 349.2 ± 14.1	61 349.8 ± 15.3	61 21.5 ± 4.0	61 21.0 ± 2.0	61 52.0 ± 4.9	61 52.1 ± 4.9	61 43.8 ± 3.2	61 43.5 ± 3.9	61 42.6 ± 4.0	61 43.4 ± 3.9	61 34.0 ± 8.7	61 32.8 ± 7.2	61 73.6 ± 11.9	61 74.7 ± 11.2	61 15.6 ± 1.7	61 15.5 ± 1.7
5 years																
T1DM	45 348.5 ± 17.5	47 348.9 ± 17.8	45 21.6 ± 2.1	47 21.5 ± 2.3	45 53.0 ± 5.4	47 53.3 ± 5.1	45 44.2 ± 3.9	47 44.1 ± 3.6	45 42.5 ± 3.5	47 43.8 ± 8.3	45 31.2 ± 5.0	47 32.7 ± 6.6	45 73.8 ± 9.1	47 71.9 ± 10.9	45 15.4 ± 1.3	47 14.9 ± 1.3
T2DM	69 339.8 ± 17.2	66 342.0 ± 15.9	69 21.6 ± 2.2	66 22.6 ± 4.6	69 49.1 ± 5.8	66 50.2 ± 5.1	69 41.2 ± 3.8	66 41.9 ± 3.4	69 41.7 ± 4.2	66 41.8 ± 4.1	69 32.5 ± 7.9	66 31.3 ± 5.8	69 72.2 ± 10.9	66 73.9 ± 10.2	69 15.4 ± 1.6	66 15.2 ± 1.5
10 years																
T1DM	37 350.9 ± 16.8	37 351.8 ± 15.1	37 21.2 ± 1.9	37 21.8 ± 2.5	37 52.8 ± 4.6	37 52.5 ± 6.0	37 43.3 ± 2.8	37 43.4 ± 3.6	37 41.5 ± 5.2	37 42.5 ± 3.7	37 35.9 ± 7.8	37 35.5 ± 7.9	37 72.5 ± 12.7	37 73.2 ± 11.8	37 15.9 ± 1.9	37 15.6 ± 1.7
T2DM	51 340.1 ± 21.8	49 341.9 ± 20.1	51 21.3 ± 2.1	49 21.5 ± 2.3	51 48.4 ± 7.5	49 48.6 ± 7.0	51 41.5 ± 4.3	49 41.2 ± 4.8	51 42.5 ± 4.3	49 42.7 ± 4.1	51 33.1 ± 7.5	49 31.7 ± 6.9	51 73.6 ± 13.9	49 74.3 ± 13.3	51 15.1 ± 1.6	49 15.4 ± 1.8

Data are presented as sample size, mean ± SD. All measurements are given in µm

LE, left eye; RE, right eye; T1DM, type 1 diabetes; T2DM, type 2 diabetes

### Statistical analysis

We modelled PD, VD, CC FD, FAZ parameters, VA, CS and retinal layer thickness separately as linear functions of age, sex, diabetes type (type 1 and 2 diabetes, no diabetes as control participants) as well as diabetes clusters, known diabetes duration (15 years of follow-up are not shown due to the small number of participants), as well as the appearance of MAs using mixed linear models (PROC MIXED). The influence of HbA_1c_ levels and blood pressure on OCTA parameters was analysed by classifying HbA_1c_ as a categorical variable (>59 mmol/mol or >7.5% vs ≤59 mmol/mol or ≤7.5%) and systolic, diastolic and mean arterial blood pressure as continuous variables. In the case of quantitative variables, *p* values were calculated via Kruskal–Wallis test for comparison of three groups (Table 2) and Mann–Whitney *U* test for comparison of two groups (Table 1). For categorical variables, *p* values are related to χ^2^ test or Fisher’s exact test if possible (Table 1).

Fisher’s exact test calculated the association of the presence of MAs with HbA_1c_ level and blood pressure. A logistic regression model with random intercept (PROC GLIMMIX) was used to analyse the effect of diabetes duration on MAs in SCP, DCP and ICP separately, adjusted for age, sex and diabetes type. In all models, dependence between measurements on two eyes of the same person was implemented via a compound symmetry structure.

The significance level was set to α=0.05. Statistical analyses were performed with SAS software, version 9.4 (SAS Institute, Cary, NC, USA).

## Results

### OCTA analysis of vascular parameters

At baseline, individuals with type 2 diabetes showed a reduced PD (expected difference [ED] compared with control individuals: −0.0071, *p*=0.0276; Table 8) and VD (ED: −0.0034, *p*=0.0184) in the SCP compared with control individuals. This effect on PD and VD of the SCP remained even after adjusting for systolic (*p*=0.0269, *p*=0.0217) and diastolic blood pressure (*p*=0.0293, *p*=0.0203, respectively; electronic supplementary material [ESM] Tables 1, 2) as well as HbA_1c_ (*p*=0.0488, *p*=0.0197, respectively; ESM Table 3).

**Table 8:** PD/VD of the SCP as dependent variable

Effect	PD of SCP		VD of SCP	
	ED (95% CI)	*p* value	ED (95% CI)	*p* value
Age (year)	−0.0005 (−0.0007, −0.0003)	<0.0001	−0.0002 (−0.0003, −0.0001)	<0.0001
Sex (male vs female)	0.0066 (0.0023, 0.0109)	0.0026	0.0013 (−0.0006, 0.0033)	0.1750
Diabetes type (1 vs no diabetes)	−0.0031 (−0.0099, 0.0038)	0.3792	−0.0015 (−0.0046, 0.0016)	0.3407
Diabetes type (2 vs no diabetes)	−0.0071 (−0.0133, −0.0008)	0.0276	−0.0034 (−0.0062, −0.0006)	0.0184
Diabetes type (1 vs 2)	0.0040 (−0.0017, 0.0097)	0.1678	0.0019 (−0.0007, 0.0045)	0.1435
Known diabetes duration (Y5 vs 0)	0.0008 (−0.0050, 0.0066)	0.7856	0.0008 (−0.0018, 0.0034)	0.5472
Known diabetes duration (Y10 vs 0)	−0.0068 (−0.0132, −0.0004)	0.0365	−0.0027 (−0.0056, 0.0001)	0.0618

Mixed-effects model

Values with *p*≤0.05 are considered statistically significant

Y5–Y10, years of follow-up

Considering heterogeneity among diabetes subtypes, a significant difference in PD and VD of the SCP was found between the diabetes subgroups SIDD and MOD compared with SAID (Table 9). MOD had reduced PD and VD of the SCP compared with MARD (*p*=0.0232, *p*=0.0237, respectively). SIDD showed lower PD of the SCP compared with all other subtypes (vs SIRD *p*=0.0073, vs MOD *p*=0.0412, vs MARD *p*=0.0036). No subtype-related difference in PD between ICP and DCP was found (Table 10). A reduced PD of SCP (*p*=0.0365; Table 8) and DCP was observed in individuals with 10 years of diabetes (*p*=0.0062; Table 11).

**Table 9:** PD/VD of the SCP as dependent variable, adjusted for age and diabetes duration

Effect	PD of SCP		VD of SCP	
	ED (95% CI)	*p* value	ED (95% CI)	*p* value
SIDD (SIDD vs SAID)	−0.0264 (−0.0431, −0.0096)	0.0021	−0.0089 (−0.0163, −0.0015)	0.0189
SIRD (SIRD vs SAID)	−0.0003 (−0.0116, 0.0111)	0.9629	−0.0020 (−0.0071, 0.0030)	0.4250
MOD (MOD vs SAID)	−0.0089 (−0.0156, −0.0021)	0.0100	−0.0050 (−0.0080, −0.0020)	0.0011
MARD (MARD vs SAID)	−0.0009 (−0.0087, 0.0068)	0.8168	−0.0015 (−0.0049, 0.0020)	0.4022
Known diabetes duration (Y5 vs 0)	0.0018 (−0.0043, 0.0078)	0.5660	0.0011 (−0.0016, 0.0037)	0.4324
Known diabetes duration (Y10 vs 0)	−0.0073 (−0.0142, −0.0005)	0.0347	−0.0031 (−0.0062, −0.0001)	0.0412
Age	−0.0005 (−0.0007, −0.0002)	0.0001	−0.0002 (−0.0003, −0.0001)	0.0040

Mixed-effects model

Values with *p*≤0.05 are considered statistically significant

Y5–Y10, years of follow-up

**Table 10:** PD of the ICP and DCP as dependent variable

Effect	PD of ICP		PD of DCP	
	ED (95% CI)	*p* value	ED (95% CI)	*p* value
SIDD (SIDD vs SAID)	−0.0140 (−0.0358, 0.0078)	0.2077	−0.0090 (−0.0407, 0.0226)	0.5747
SIRD (SIRD vs SAID)	0.0003 (−0.0146, 0.0151)	0.9727	0.0102 (−0.0116, 0.0320)	0.3575
MOD (MOD vs SAID)	−0.0011 (−0.0099, 0.0077)	0.8064	−0.0005 (−0.0133, 0.0123)	0.9376
MARD (MARD vs SAID)	0.0008 (−0.0093, 0.0109)	0.8812	0.0087 (−0.0061, 0.0234)	0.2475
Known diabetes duration (Y5 vs 0)	−0.00140 (−0.0093, 0.0065)	0.7267	−0.0003 (−0.0117, 0.0112)	0.9617
Known diabetes duration (Y10 vs 0)	−0.00490 (−0.0138, 0.0040)	0.2788	−0.0170 (−0.0301, −0.0040)	0.0107
Age	−0.0010 (−0.0012, −0.0006)	<0.0001	−0.0012 (−0.0017, −0.0007)	<0.0001

Mixed-effects model

Values with *p*≤0.05 are considered statistically significant

Y5–Y10, years of follow-up

**Table 11:** PD of the DCP/ICP as dependent variable and age, sex, diabetes type and diabetes duration as independent variables

Effect	PD of DCP		PD of ICP	
	ED (95% CI)	*p* value	ED (95% CI)	*p* value
Age	−0.0013 (−0.0016, −0.0001)	<0.0001	−0.0009 (−0.0011, −0.0007)	<0.0001
Sex (male vs female)	0.0076 (−0.0009, 0.0160)	0.0805	0.0024 (−0.0028, 0.0076)	0.3732
Diabetes type (1 vs no diabetes)	−0.0074 (−0.0209, 0.0060)	0.2790	−0.0040 (−0.0123, 0.0042)	0.3380
Diabetes type (2 vs no diabetes)	0.0014 (−0.0110, 0.0137)	0.8277	−0.0043 (−0.0119, 0.0033)	0.2647
Known diabetes duration (Y5 vs 0)	−0.0009 (−0.0123, 0.0105)	0.8749	−0.0015 (−0.0086, 0.0055)	0.6664
Known diabetes duration (Y10 vs 0)	−0.0177 (−0.0303, −0.0051)	0.0062	−0.0056 (−0.0133, 0.0022)	0.1573

Mixed-effects model. Data for 15 years of follow-up are not shown due to the small number of participants

Values with *p*≤0.05 are considered statistically significant

Y5–Y10, years of follow-up

An association between PD across all plexuses and diabetes duration was found (*p*<0.05). In contrast, no clear association between HbA_1c_ and PD was observed in the SCP (left eye: *p*=0.0520, right eye: *p*=0.0574).

The PD of the SCP, ICP and DCP as well as the VD of the SCP decreased with age (Tables 8, 9, 10 and 11). The PD of SCP was higher in men (*p*=0.0026) and the FAZ was larger in female participants (*p*=0.0001; Table 12), indicating sex differences. The FAZ was greater in both type 1 diabetes (*p*=0.0327) and type 2 diabetes (*p*=0.0006) compared with healthy participants. Individuals with diabetes had lower FAZ circularity and a larger perimeter, more pronounced in type 2 (both *p*=0.0001; Table 12), especially in the MARD and MOD subgroups (ESM Table 4).

**Table 12:** FAZ area, FAZ perimeter and FAZ circularity of the SCP as dependent variable

Effect	FAZ area		FAZ perimeter		FAZ circularity	
	ED (95% CI)	*p* value	ED (95% CI)	*p* value	ED (95% CI)	*p* value
Age (year)	0.0002 (−0.0011, 0.0015)	0.7721	−0.0054 (−0.0186, 0.0078)	0.4227	0.0012 (−0.0001, 0.0025)	0.0590
Sex (male vs female)	−0.0689 (−0.1013, −0.0365)	<0.0001	−0.2534 (−0.5833, 0.0765)	0.1317	−0.0254 (−0.0575, 0.0067)	0.1210
Diabetes type (1 vs no diabetes)	0.0552 (0.0046, 0.1059)	0.0327	1.1039 (0.5889, 1.6189)	<0.0001	−0.0829 (−0.1331, −0.0328)	0.0013
Diabetes type (2 vs no diabetes)	0.0819 (0.0355, 0.1283)	0.0006	1.6052 (1.1322, 2.0782)	<0.0001	−0.1358 (−0.1819, −0.0897)	<0.0001
Diabetes type (1 vs 2)	−0.0267 (−0.0684, 0.0150)	0.2085	−0.5013 (−0.9248, −0.0778)	0.0205	0.0529 (0.0116, 0.0941)	0.0121
Known diabetes duration (Y5 vs 0)	−0.0160 (−0.0588, 0.0268)	0.4635	−0.3569 (−0.7920, 0.0781)	0.1075	0.0028 (−0.0396, 0.0452)	0.8968
Known diabetes duration (Y10 vs 0)	−0.0128 (−0.0595, 0.0338)	0.5884	−0.4567 (−0.9311, 0.0178)	0.0592	0.0151 (−0.0311, 0.0614)	0.5198
Mean arterial pressure	0.0002 (−0.0012, 0.0017)	0.7531	0.0096 (−0.0049, 0.0241)	0.1949	−0.0011 (−0.0025, 0.0003)	0.1252

Mixed-effects model

Values with *p*≤0.05 are considered statistically significant

Y5–Y10, years of follow-up

FAZ and CC FD negatively correlated with PD in the SCP, ICP and DCP (ESM Table 5).

Participants with both type 1 and, in particular, type 2 diabetes exhibited higher CC FD than control individuals (*p*=0.0474, *p*<0.0001, respectively). Men had lower CC FD than women (Table 13).

**Table 13:** Choriocapillaris flow deficit area as dependent variable

Effect	Choriocapillaris flow deficit area (%)	
	ED (95% CI)	*p* value
Age (year)	0.2372 (0.1799, 0.2946)	<0.0001
Sex (male vs female)	−2.3573 (−3.8116, −0.9031)	0.0016
Diabetes type (1 vs no diabetes)	2.2775 (0.0265, 4.5286)	0.0474
Diabetes type (2 vs no diabetes)	4.8155 (2.7404, 6.8907)	<0.0001
Diabetes type (1 vs 2)	−2.5380 (−4.4044, −0.6716)	0.0079
Known diabetes duration (Y5 vs 0)	−0.0273 (−1.9081, 1.8535)	0.9772
Known diabetes duration (Y10 vs 0)	−0.4354 (−2.4836, 1.6128)	0.6761
**Mean arterial pressure**	0.0619 (−0.0015, 0.1253)	0.0554

Mixed-effects model

Values with *p*≤0.05 are considered statistically significant

Y5–Y10, years of follow-up

Overall, MAs were found in 12.0% of individuals in the SCP (2.7% in type 1, 9.0% in type 2 diabetes), 14.4% in the ICP (3.3% in type 1, 10.8% in type 2 diabetes) and 10.2% in the DCP (2.4% in type 1, 7.8% in type 2 diabetes). One control participant (0.3%) showed MAs in the ICP and DCP. Of the 170 MAs (51 MAs in DCP, 69 MAs in ICP and 50 MAs in SCP) detected by OCTA in 57 individuals, only 11 (9%) exhibited corresponding findings on non-mydriatic fundus images. MAs in the SCP and DCP were significantly more prevalent after 5 (*p*=0.0209, *p*=0.0279, respectively) and 10 years (*p*=0.0220, *p*=0.0007) of diabetes, while the occurrence of MAs in the ICP was more frequent after 10 years, compared with baseline (Table 14).

**Table 14:** Presence of MAs in the DCP, ICP and SCP as dependent variable and known diabetes duration, diabetes type, age and sex as influencing factors

Effect	OR (95% CI)	*p* value
MAs in DCP		
Age	1.01 (0.98, 1.04)	0.4531
Sex (female vs male)	0.71 (0.35, 1.46)	0.3540
Diabetes type (1 vs 2)	0.65 (0.27, 1.58)	0.3385
Diabetes duration (5 vs 0)	3.52 (1.15, 10.81)	0.0279
Diabetes duration (10 vs 0)	6.98 (2.27, 21.47)	0.0007
MAs in ICP		
Age	1.03 (1.0, 1.06)	0.0893
Sex (female vs male)	0.75 (0.39, 1.46)	0.4023
Diabetes type (1 vs 2)	0.75 (0.33, 1.71)	0.4891
Diabetes duration (5 vs 0)	1.72 (0.74, 4.00)	0.2045
Diabetes duration (10 vs 0)	2.43 (1.02, 5.81)	0.0461
MAs in SCP		
Age	1.00 (0.96, 1.04)	0.9831
Sex (female vs male)	0.83 (0.35, 1.98)	0.6692
Diabetes type (1 vs 2)	0.43 (0.13, 1.38)	0.1537
Diabetes duration (5 vs 0)	4.45 (1.26, 15.74)	0.0209
Diabetes duration (10 vs 0)	4.78 (1.26, 18.23)	0.0220

GLIMMIX model

Values with *p*≤0.05 are considered statistically significant

The detection of MAs in all retinal plexuses was associated with thinning of the GCL, specifically in the SCP (ED by manifestation of MAs=−1.95 µm, *p*=0.0129). Detection of MAs in the ICP is associated with a thinner GCL (1.72 µm, *p*=0.0069). MAs in the DCP were associated with a thinner GCL (2.14 µm, *p*=0.0008) and a low IPL thickness (−1.28, *p*=0.0044), while MAs in the SCP were linked to thin ONL (−3.70 µm, *p*=0.0312; Table 15). Besides, MAs in the SCP and DCP were associated with reduced PD in both the SCP and ICP, and decreased VD in the SCP and DCP (Table 16). MAs located in the ICP were associated with decreased PD there (*p*=0.0423). Furthermore, the occurrence of MAs in the DCP and the HbA_1c_ value were moderately linked (Fisher’s exact test: *p*=0.0044). Moreover, higher systolic and diastolic blood pressure were associated with the presence of MAs in the DCP (*p*=0.0199 and *p*=0.0040, respectively), while high diastolic blood pressure was also linked to the presence of MAs in the SCP and ICP (*p*=0.0126 and *p*=0.0166, respectively).

**Table 15:** GCL, IPL and ONL as dependent variables and the presence of MAs in the different plexuses as independent variables

Effect	GCL		IPL		ONL	
	ED (95% CI)	*p* value	ED (95% CI)	*p* value	ED (95% CI)	*p* value
MAs in DCP	−2.1420 (−3.3045, −0.9796)	0.0008	−1.2808 (−2.1243, −0.4373)	0.0044	−1.8272 (−4.5746, 0.9202)	0.1833
MAs in ICP	−1.7215 (−2.9194, −0.5235)	0.0069	−0.7873 (−1.6490, 0.0744)	0.0713	−2.2821 (−5.0042, 0.4399)	0.0961
MAs in SCP	−1.9482 (−3.4211, −0.4753)	0.0129	−0.7070 (−1.7647, 0.3507)	0.1747	−3.6972 (−7.0132, −0.3811)	0.0312

Mixed-effects models; adjustments were made for age, sex, diabetes type and diabetes duration

Values with *p*≤0.05 are considered statistically significant

**Table 16:** PD of the SCP, ICP and DCP and VD of the SCP as dependent variables and the presence of MAs in the different plexuses as independent variables

Effect	PD in SCP		VD in SCP		PD in DCP		PD in ICP	
	ED (95% CI)	*p* value	ED (95% CI)	*p* value	ED (95% CI)	*p* value	ED (95% CI)	*p* value
MAs in DCP	−0.0082 (−0.0157, −0.0008)	0.0316	−0.0035 (−0.0067, −0.0002)	0.0368	−0.0064 (−0.0239, 0.0111)	0.4598	−0.0110 (−0.0200, −0.0019)	0.0192
MAs in ICP	−0.0020 (−0.0089, 0.0049)	0.5533	−0.0016 (−0.0046, 0.0014)	0.2829	−0.0038 (−0.0191, 0.0115)	0.6141	−0.0086 (−0.0169, −0.0003)	0.0423
MAs in SCP	−0.0131 (−0.0214, −0.0049)	0.0039	−0.0058 (−0.0095, −0.0022)	0.0037	−0.0123 (−0.0307, 0.0061)	0.1754	−0.0150 (−0.0250, −0.0050)	0.0058

Mixed-effects model; adjustments were made for age, sex, diabetes type and diabetes duration

Values with *p*≤0.05 are considered statistically significant

### Structural OCT analysis

Total retinal thickness decreased with age (annual ED: −0.4 µm, *p*<0.0001) and was thicker in male than in female individuals (ED: +6.3 µm, *p*=0.0001). Thus, there was an age-related thinning of the layer thickness of the GCL (ED: −0.2 µm, *p*<0.0001), IPL (ED: −0.1 µm, *p*<0.0001), OPL (ED: −0.05 µm, *p*=0.0491), ONL (ED: −0.1 µm, *p*=0.0140) and RPE (ED: −0.02 µm, *p*=0.0103). Male individuals also showed a thicker RNFL (ED: +0.6 µm, *p*=0.0070), ONL (ED: +2.8 µm, *p*=0.0092) and INL (ED: +2.2 µm, *p*<0.0001). Diabetes duration had no impact on total retinal thickness up to 10 years after diagnosis.

The diabetes subtype analysis showed lower RPE thickness for people with SIDD than for those with SAID (ED=−1.1192 µm, *p*=0.0491).

### VA and CS

VA and CS showed a decrease with increasing age (*p*<0.0001 for both; ESM Table 6). Lower CS values were found in male participants (compared with women, *p*=0.0317) and with higher systolic blood pressure (*p*=0.0395). At 10 year follow-up, VA was reduced in individuals with diabetes (*p*=0.0434).

## Discussion

This study showed that clinically invisible microvascular changes of PD, VD and FAZ can be detected by OCTA in type 2 diabetes, especially in participants with SIDD and MOD, with a known duration up to 1 year, without any evidence of diabetic retinal neurodegeneration or functional deficits. Moreover, our findings indicate that MAs and signs of retinal neurodegeneration occur simultaneously.

### Retinal microvascular parameters

Consistent with other publications, we found an association between diabetes and reduced PD before clinical signs of diabetic retinopathy appeared [6, 21–24]. However, while reduced superficial macular capillary flow has previously been observed after more than a year of type 2 diabetes, we noted reduced PD and VD even within the first year after diagnosis.

Lee et al found reduced VD in the SCP in individuals with type 2 diabetes with a mean disease duration of 3.5 years [21]. Tan et al showed a reduced retinal capillary density in the macular SCP and DCP in individuals after a mean duration of type 2 diabetes of 5.5 years [22]. Vujosevic et al demonstrated microvascular changes, including capillary loss in both the superficial and deep plexus, in individuals with type 1 diabetes after 16.3 years and in those with type 2 diabetes after 7.7 years, as detected by OCTA [24].

Another important finding from our data is that the three levels of the capillary plexus are affected to varying degrees by diabetes. The earliest diabetes-related alterations are found in the choriocapillaris and SCP, and later in the DCP. The ICP is not involved within up to 10 years. An anatomical reason could be that the intermediate plexus is not always clearly defined [25].

Consistent with other publications, an enlarged FAZ, increased FAZ perimeter and reduced FAZ circularity in the SCP were observed in individuals with diabetes without diabetic retinopathy compared with healthy control individuals [26]. This increase in FAZ was detectable within the first year of the disease and was accompanied by reduced SCP perfusion, consistent with other studies [27]. Capillary occlusions are suspected to be the most likely cause of the increase in FAZ in diabetes [28].

We were able to demonstrate that the SIDD subtype, known for its high risk of developing diabetic retinopathy [13], and MOD, characterised by obesity and moderate insulin resistance [14], are more likely to exhibit microvascular changes in the SCP compared with SAID. In SIDD, the microvascular retinal changes in the SCP are different in comparison with all other clusters. This effect is intriguing as SIDD closely resembles metabolic features of type 1 diabetes, except for the absence of islet-directed antibodies. The older age at diagnosis in SIDD compared with type 1 diabetes, along with a relatively later start of insulin therapy and persistent hyperglycaemia, may explain the higher frequency of retinal microangiopathy observed in SIDD.

No early changes in VD or PD in the SCP were observed in type 1 diabetes, but a smaller FAZ perimeter and greater circularity were found compared with type 2 diabetes. This may be explained by both the lower age and lower rates of comorbidities such as hypertension, dyslipidaemia and obesity, which are known risk factors of endothelial dysfunction [29, 30]. In our cohort, individuals with type 1 diabetes showed a lower prevalence of hypertension as well as lower BMI (Table 1). However, the effect on perfusion remained statistically significant even after adjustment for these parameters. Moreover, individuals with type 2 diabetes are more likely to initially develop diabetic macular oedema than those with type 1 diabetes [31], while those with type 1 diabetes are more prone to diabetic retinopathy [32]. This could be explained by the longer asymptomatic phase of dysglycaemia before diagnosis and the associated longer exposure to metabolic dysregulation in type 2 diabetes with higher susceptibility to macular vascular damage [29]. Interestingly, Fleissig et al demonstrated a higher rate of crossing vessels through the fovea and a less severe FAZ in individuals with type 1 diabetes compared with those with type 2 diabetes, consistent with our findings. It is conceivable that individuals with type 1 diabetes release early protective factors that may lead to secondary capillary remodelling [33].

The altered choriocapillary perfusion observed early in type 1 and type 2 diabetes, as shown here and by others [27], may correspond to choriocapillaris occlusions caused by hyperglycaemia-induced leukostasis and inflammation [34].

### MAs

Widefield swept-source OCTA is comparable to ultra-widefield fluorescein angiography in the detection rate of MAs, intraretinal microvascular abnormalities and neovascularisations [35]. Moreover, OCTA allows the detection of early ophthalmoscopically undetected retinal diabetic changes [36]. We showed that only 9% of the MAs detected by OCTA were visible in the fundus photography.

Another novel association we found was reduced GCL and IPL thickness as well as reduced PD and VD in individuals with diabetes when MAs were detectable on OCTA. Reduced perfusion in the SCP and ICP may be responsible for the thinning of GCL and IPL, which is described as the earliest neurodegenerative change before the onset of diabetic retinopathy [37, 38]. Qiu et al showed that the GCL cell complex correlates with the VD of SCP and DCP as well as the severity of diabetic retinopathy, suggesting an interaction between retinal microvasculopathy and neuronal degeneration [39]. We found no relationship between MAs in the DCP and the thickness of the INL and OPL, which are anatomically located at the same level [38].

Another important observation is the association between non-invasive detection of MAs using OCTA and blood pressure, which reflects perfusion disturbances and thus provides insights into cardiovascular and metabolic status. As Yao et al, we observed reduced PD and VD in the SCP of individuals with diabetes, but without evidence of an influence of blood pressure on retinal microcirculation [40]. One explanation could be that our cohort consisted of individuals highly motivated to adhere to the therapy recommendations [12].

### Retinal neurodegenerative alterations

Several studies have indicated that the retinal neurodegenerative component already occurs before the onset of diabetic retinopathy substantiated by retinal thinning of the inner retina including the GCL, RNFL and IPL, and functional deficits demonstrated by colour vision deficits, delayed dark adaptation, visual field defects, poorer CS and abnormal multifocal electroretinogram [41–43]. A longitudinal study investigating neurodegenerative changes in the macular retinal layers in type 1 diabetes demonstrated a significant decrease in RNFL and GCL [44].

We recently demonstrated that individuals from the GDS baseline cohort showed no signs of neurodegenerative damage to the retina as detected by retinal thickness measurements using spectral-domain optical coherence tomography [15]. However, we now have evidence of neurodegeneration, as measured by reduced GCL, IPL and ONL in individuals with diabetes when MAs are present on OCTA. Since MAs were found more frequently after 5 and especially after 10 years, the neurodegenerative changes appear to be time-dependent and linked to vascular changes (Tables 14, 15). Otherwise, specific characteristics of our study cohort as a highly motivated and informed group with conscious disease management and exclusion of participants with very high HbA_1c_ levels could account for the overall lack of evidence of general thinning of retinal inner layers. The resulting improved treatment adherence may prevent neurodegeneration [12].

Another interesting observation emerged from our diabetes subtype analysis, indicating that the SIDD subtype has lower RPE thickness than individuals with SAID. According to clinical and animal data, RPE damage in the early stages of diabetes could serve as an early biomarker for disease progression [45]. Consistent with our results, Yang et al found that RPE thickness in type 2 diabetes is associated with the risk of the microvascular phenotype (diabetic retinopathy, macular capillary loss) [45]. It is possible that the relatively late start of insulin treatment and metabolic control explains the higher risk of diabetic retinopathy and polyneuropathy [13], initially manifested by reduced capillary density in the SCP or RPE dysfunction, and, as we show, reduced choriocapillary flow.

### Physiological effects

Consistent with previous studies, we found progressive thinning with age of total retinal thickness, and of GCL, IPL, ONL and RPE, and larger values of total retinal thickness, INL, ONL, RNFL and PD in male participants [46–48]. CS decreases with age and, in contrast to the literature, we found that CS is lower in male participants [49]. This finding might be consistent with the hypothesis that women tend to perform better in near vision, while men typically perform better in accurately perceiving and estimating the size of distant objects (hunter–gatherer hypothesis) [50].

Men exhibited a smaller FAZ than women, likely due to their greater central retinal thickness observed in our study. When the study was designed, it was decided to determine the gender of participants through self-report. This could result in discrepancies between self-assessment and biological sex and, under certain circumstances, comparability with studies that determined biological sex could be limited.

### Limitations

As the primary aim of this study was to investigate early diabetes-associated changes in the capillary system of the macula, a cross-sectional design was used, which inherently limits the study. To rule out early functional neurodegeneration, it would be ideal to include multifocal electroretinography, along with CS assessments, which was not feasible in this large-scale study. Selection bias cannot be ruled out when evaluating the GDS cohort [12].

The heterogeneous OCTA data in diabetic retinopathy result from inconsistent nomenclature, varying quantification of OCT angiograms, OCT manufacturer-dependent algorithms, lateral resolution, scanning modes and different angiocube scan size, even with the same device [6].

A strength of the present study is the use of only one type of OCT/OCTA device and the combined approach of OCTA and OCT to evaluate neurodegenerative and microvascular changes. Another strength is the prospective long-term follow-up, including sophisticated metabolic phenotyping and quantitative assessment of diabetes-related chronic complications and comorbidities in all participants [12]. This advantage will be used to assess retinal microvascular and neurodegenerative changes in a longitudinal analysis in the future.

### Conclusions

In the early stages of type 2 diabetes, particularly in individuals with SIDD and MOD, OCTA can detect alterations in capillary perfusion in the SCP of the macula. With longer disease duration, such alterations extend to both the SCP and DCP in type 1 and type 2 diabetes. Additionally, early alterations in the choriocapillaris in both diabetes types could serve as a potential early marker of the disease. Furthermore, OCTA-detected MAs, associated with thinning of the GCL and IPL and reduced PD in the SCP and ICP, may serve as biomarkers for preclinical retinal neurovascular damage. This study suggests that the SIDD subtype, characterised by early reduction in RPE thickness and microvascular changes, may represent the microvascular phenotype of type 2 diabetes.

All in all, these findings emphasise OCTA’s ability to detect early choroidal vasculopathy, reduced retinal perfusion and MAs associated with neurovascular alterations—key changes that occur before a decline in VA. Consequently, OCTA parameters could enable sensitive and comprehensive assessment of neurovascular interactions in the retina and potentially serve as endpoints for future intervention studies in preclinical diabetic retinopathy.

## Supplementary Information

Below is the link to the electronic supplementary material.

ESM Tables (PDF 179 KB)

## Data Availability

All data supporting the findings of this study are available within the paper and its electronic supplementary material (ESM). The datasets are available from the corresponding author upon reasonable request and after discussion of the request within the GDS steering committee. The study protocol and the individual methods have been published in the cohort profile [12] and are unrestrictedly available.

## Abbreviations

CC FD: Choriocapillaris flow deficit density
CS: Contrast sensitivity
DCP: Deep capillary plexus
ED: Expected difference
ETDRS: Early Treatment Diabetic Retinopathy Study
FAZ: Foveal avascular zone
GCL: Ganglion cell layer
GDS: German Diabetes Study
ICP: Intermediate capillary plexus
INL: Inner nuclear layer
IPL: Inner plexiform layer
MA: Microaneurysm
MARD: Mild age-related diabetes
MOD: Mild obesity-related diabetes
OCT: Optical coherence tomography
OCTA: Optical coherence tomography angiography
ONL: Outer nuclear layer
OPL: Outer plexiform layer
PD: Perfusion density
RNFL: Retinal nerve fibre layer
RPE: Retinal pigment epithelium
SAID: Severe autoimmune diabetes
SCP: Superficial capillary plexus
SIDD: Severe insulin-deficient diabetes
SIRD: Severe insulin-resistant diabetes
VA: Visual acuity
VD: Vessel density

## Authors' relationships and activities

SK has participated in the scientific advisory board of Johnson & Johnson. GG has participated in consulting for and/or scientific advisory boards of Santen, Holostem/Chiesi, Novartis, Thea Pharma and Med1 Ventures. PS received speaker honoraria from Bayer. MR received fees for consulting, lecturing or serving on advisory boards from Astra Zeneca, Boehringer Ingelheim, Echosens, Eli Lilly, Merck-MSD, Madrigal, Novo Nordisk, Madrigal, Synlab and Target RWE, and has performed investigator-initiated research with support from Boehringer Ingelheim and Novo Nordisk to the DDZ. The research of MR is also supported by grants from the German Research Foundation (DFG; RTG/GRK 2576), the European Community (HORIZON-HLTH-2022-STAYHLTH-02-01: Panel A) to the INTERCEPT-T2D consortium and the Schmutzler-Stiftung. RG has participated in consulting for and/or scientific advisory boards of BayerHealthCare, RochePharmaAG and Abbvie.
