# Supplementary material for: Macular perfusion alterations in people with recent-onset diabetes and novel diabetes subtypes
Source: Diabetologia. 2025 Mar 31;68(6):1140–56. doi: 10.1007/s00125-025-06407-5 (PMC12069482; doi:10.1007/s00125-025-06407-5)
Supplement: Supplementary file 1 — ESM Tables (PDF 179 KB) [file 125_2025_6407_MOESM1_ESM.pdf]

Macular perfusion alterations in people with recent-onset diabetes and novel diabetes subtypes
Electronic supplementary material

ESM Table 1: PD/VD of the SCP as dependent variable, adjusted for systolic blood pressure

Effect	PD of SCP			VD of SCP	
	Expected difference (95% CI)	*p* value		Expected difference (95% CI)	*p* value
Age (year)	-0.0005 (-0.0007, -0.0003)	<0.0001		-0.0002 (-0.0003, -0.0001)	<0.0001
Sex (male vs female)	0.0068 (0.0023, 0.0113)	0.0033		0.0011 (-0.0009, 0.0031)	0.2869
Diabetes type (1 vs no diabetes)	-0.0025 (-0.0094, 0.0044)	0.4774		-0.0013 (-0.0044, 0.0019)	0.4311
Diabetes type (2 vs no diabetes)	-0.0071 (-0.0134, -0.0008)	0.0269		-0.0033 (-0.0062, -0.0005)	0.0217
Diabetes type (1 vs 2)	0.0046 (-0.0011, 0.0104)	0.1136		0.0021 (-0.0005, 0.0047)	0.1135
Known diabetes duration (Y5 vs 0)	0.0005 (-0.0054, 0.0064)	0.8655		0.0006 (-0.0020, 0.0033)	0.6381
Known diabetes duration (Y10 vs 0)	-0.0066 (-0.0130, -0.0001)	0.0453		-0.0028 (-0.0057, 0.0001)	0.0576
Systolic blood pressure	-5.05×10^{-6} (-0.0001, 0.00013)	0.9417		0.00002 (-0.0000, 0.0001)	0.5289

Mixed-effects model, Values with $p \leq 0.05$ are considered statistically significant, Y5–Y10, years of follow-up

ESM Table 2: PD/ VD of the SCP as dependent variable, adjusted for diastolic blood pressure

Effect	PD of SCP			VD of SCP	
	Expected difference (95% CI)	*p* value		Expected difference (95% CI)	*p* value
Age (year)	-0.0005 (-0.0007, -0.0003)	<0.0001		-0.0002 (-0.0003, -0.0001)	<0.0001
Sex (male vs female)	0.0069 (0.0025, 0.0112)	0.0021		0.0012 (-0.0008, 0.0032)	0.2391
Diabetes type (1 vs no diabetes)	-0.0025 (-0.0094, 0.0044)	0.4737		-0.0013 (-0.0044, 0.0018)	0.4124
Diabetes type (2 vs no diabetes)	-0.0070 (-0.0134, -0.0007)	0.0293		-0.0034 (-0.0062, -0.0005)	0.0203
Diabetes type (1 vs 2)	0.0045 (0.0012, 0.0103)	0.1220		0.0021 (-0.0005, 0.0047)	0.1140
Known diabetes duration (Y5 vs 0)	0.0006 (-0.0053, 0.0065)	0.8385		0.0006 (-0.0020, 0.0033)	0.6376
Known diabetes duration (Y10 vs 0)	-0.0067 (-0.0132, -0.0003)	0.0414		-0.0027 (-0.0056, 0.0002)	0.0696
Diastolic blood pressure	-0.0001 (-0.0003, 0.0002)	0.6213		0.00003 (-0.0001, 0.0001)	0.5140

Mixed-effects model, Values with $p \leq 0.05$ are considered statistically significant, Y5–Y10, years of follow-up

ESM Table 3: PD/ VD of the SCP as dependent variable, adjusted for HbA1c

Effect	PD of SCP			VD of SCP	
	Expected difference (95% CI)	*p* value		Expected difference (95% CI)	*p* value
Age (year)	-0.0005 (-0.0007, -0.0003)	<0.0001		-0.0002 (-0.0003, -0.0001)	<0.0001
Sex (male vs female)	0.0072 (0.0028, 0.0115)	0.0013		0.0011 (-0.0004, 0.0036)	0.3028
Diabetes type (1 vs no diabetes)	-0.0024 (-0.0093, 0.0045)	0.4907		-0.0012 (-0.0043, 0.0019)	0.4332
Diabetes type (2 vs no diabetes)	-0.0064 (-0.0128, -0.00003)	0.0488		-0.0034 (-0.0060, -0.0002)	0.0197
Known diabetes duration (Y5 vs 0)	0.0012 (-0.0047, 0.0070)	0.6934		0.0007 (-0.0017, 0.0036)	0.6109
Known diabetes duration (Y10 vs 0)	-0.00595 (-0.0125, 0.0006)	0.0730		-0.0028 (-0.0053, 0.0006)	0.0562
HbA_{1c}	-0.00512 (-0.0112, 0,0010)	0.0998		0.0010 (-0.0142, 0.0070)	0.3503

Mixed-effects model, Values with $p \leq 0.05$ are considered statistically significant, Y5–Y10, years of follow-up

ESM Table 4: FAZ perimeter and FAZ circularity of the SCP as dependent variable

Effect	FAZ perimeter			FAZ circularity	
	Expected difference (95% CI)	*p* value		Expected difference (95% CI)	*p* value
SIDD (SIDD vs SAID)	0.2966 (-1.1553, 1.7486)	0.6878		-0.0177 (-0.1558, 0.1203)	0.8005
SIRD (SIRD vs SAID)	-0.3456 (-1.1728, 0.4817)	0.4115		-0.0234 (-0.1019, 0.0552)	0.5585
MOD (MOD vs SAID)	0.6074 (0.1057, 1.1090)	0.0178		-0.0597 (-0.1074, -0.0120)	0.0143
MARD (MARD vs SAID)	0.5135 (0.0190, 1.0080)	0.0419		-0.0479 (-0.0949, -0.0009)	0.0457

Mixed-effects model, Y5-Y10: years of follow up. Values with p≤ 0.05 are considered statistically significant.

ESM Table 5: FAZ area and choriocapillaris flow deficit area correlates with PD and VD

Effect	Correlation with FAZ area (*p* value)			Correlation with choriocapillaris flow deficit (*p* value)	
	RE	LE		RE	LE
PD of SCP	-0.24 (<0.0001)	-0.26 (<0.0001)		-0.35 (<0.0001)	-0.46 (<0.0001)
VD of SCP	-0.26 (<0.0001)	-0.29 (<0.0001)		-0.25 (<0.0001)	-0.36 (<0.0001)
PD of ICP	-0.16 (0.0021)	-0.16 (0.0022)		-0.30 (<0.0001)	-0.38 (<0.0001)
PD of DCP	-0.23 (<0.0001)	-0.07 (0.2259)		-0.41 (<0.0001)	-0.30 (<0.0001)

Correlations (Pearson correlation coefficients) were assessed separately for the right and left eye (RE, LE). p-value is related to the null hypothesis that the true correlation is zero.

Macular perfusion alterations in people with recent-onset diabetes and novel diabetes subtypes
Electronic supplementary material

ESM Table 6: ETDRS VA and CS as dependent variable

Effect	VA			CS	
	Expected difference (95% CI)	*p* value		Expected difference (95% CI)	*p* value
Age (year)	-0.1567 (-0.2193, -0.0941)	<0.0001		-0.0049 (-0.0059, -0.004)	<0.0001
Sex (male vs female)	-1.0817 (-2.5829, 0.4195)	0.1574		-0.0250 (-0.0478, -0.0022)	0.0317
Diabetes type (1 vs no diabetes)	0.9085 (-1.4317, 3.2488)	0.4458		-0.0103 (-0.0461, 0.0256)	0.5748
Diabetes type (2 vs no diabetes)	0.0564 (-2.1647, 2.2774)	0.9602		-0.016 (-0.0499, 0.0179)	0.3547
Known diabetes duration (Y5 vs 0)	0.5410 (-1.4649, 2.5469)	0.5963		0.0179 (-0.0123, 0.0481)	0.2447
Known diabetes duration (Y10 vs 0)	-2.2726 (-4.4774, -0.0677)	0.0434		-0.00840 (-0.0417, 0.0249)	0.6197

Mixed-effects model, Y5-Y10: years of follow up. Values with p≤ 0.05 are considered statistically significant.
